# Central effects of short-term spinal cord stimulation in postherpetic neuralgia: a longitudinal fMRI and DTI study

**DOI:** 10.3389/fnins.2025.1744783

**Published:** 2026-01-13

**Authors:** Xinyu Lei, Tingting Liao, Ruilin He, Xiaoping Yu, Yansheng Qin, Xin Hu, Xiaolong Ye, Bingfeng Lu, Zongbin Jiang

**Affiliations:** 1 Department of Pain Medicine, The Second Affiliated Hospital of Guangxi Medical University, Nanning, China; 2 Department of Radiology, The Second Affiliated Hospital of Guangxi Medical University, Nanning, China

**Keywords:** diffusion tensor imaging, functional MRI, neuropathic pain, neuroplasticity, postherpetic neuralgia, spinal cord stimulation

## Abstract

**Objective:**

Postherpetic neuralgia (PHN), a refractory neuropathic pain following herpes zoster reactivation, lacks clear central mechanisms for emerging therapies like short-term spinal cord stimulation (stSCS). This longitudinal study used multimodal neuroimaging to examine the effects of 14-day stSCS on brain function and white matter microstructure in PHN patients, and to identify neural correlates of clinical improvements.

**Methods:**

In this longitudinal, single-arm, pre-post study, 17 PHN patients received 14 days of continuous stSCS. Clinical outcomes including pain intensity (Numeric Rating Scale, NRS), anxiety and depression (Hospital Anxiety and Depression Scale, HADS), and sleep quality (Pittsburgh Sleep Quality Index, PSQI), were assessed pre-stSCS and 3 days post-stSCS. Resting-state functional MRI (rs-fMRI) and Diffusion Tensor Imaging (DTI) data were acquired at both time points. Longitudinal changes in amplitude of low-frequency fluctuations (ALFF) and fractional ALFF (fALFF) were analyzed, alongside white matter integrity via TBSS and ROI analysis of key tracts.

**Results:**

Post-stSCS, significant improvements occurred in all clinical outcomes (Wilcoxon signed-rank, all *p* < 0.001). Neuroimaging showed no DTI microstructural changes but significant fALFF increases in regions including the dorsal striatum. Notably, right medial orbitofrontal cortex (mOFC) fALFF increases correlated with NRS reductions (Spearman’s *r* = 0.71, FDR-corrected *p* = 0.036). Baseline cingulum integrity (lower FA, higher MD/RD) predicted greater striatal fALFF changes (*r* = ±0.75, FDR-corrected *p* < 0.02).

**Conclusion:**

These findings suggest that stSCS’s early clinical benefits in PHN are mediated by rapid functional reorganization rather than immediate microstructural changes. This reorganization appears prominent within fronto-striatal circuits: specifically, mOFC functional changes correlate with analgesia, while baseline cingulum integrity predicts subsequent striatal plasticity. This provides initial mechanistic insights into stSCS and suggest that baseline brain structure could be explored as a potential biomarker for treatment response, warranting validation in larger, controlled cohorts.

## 1 Introduction

Postherpetic neuralgia (PHN) is a common and challenging neuropathic pain syndrome caused by reactivation of the varicella-zoster virus, with prevalence increasing steeply with age among older adults (Forbes et al., 2016). It is characterized by persistent pain lasting more than 3 months after the resolution of the herpes zoster rash, often resulting from viral-induced nerve damage that leads to central sensitization and maladaptive neuroplasticity in the brain (Johnson and Rice, 2014; Colloca et al., 2017). In addition to pain, PHN frequently co-occurs with anxiety, depression, and sleep disturbances, which further complicate clinical management and substantially reduce patients’ quality of life (Drolet et al., 2010; Sachau et al., 2023). Although guideline-recommended first-line medications such as pregabalin and gabapentin may provide symptom relief for some patients, a substantial proportion do not achieve sufficient pain relief (Finnerup et al., 2015; Cruccu and Truini, 2017). These limitations highlight the need for alternative treatment strategies and a clearer understanding of their underlying mechanisms.

Neuromodulation therapies, particularly spinal cord stimulation (SCS), have become an important treatment option for refractory PHN (Isagulyan et al., 2023). However, permanent SCS implantation faces challenges such as high costs, infection risks, and long-term maintenance issues (Eldabe et al., 2016). In recent years, short-term spinal cord stimulation (stSCS) has emerged as a minimally invasive, low-cost alternative with good patient compliance. It has demonstrated promising efficacy in treating PHN and its comorbidities, with benefits observed in short-term and long-term follow-up (Li et al., 2025; Abbas et al., 2025). Meanwhile, the development of neuroimaging techniques, such as resting-state functional magnetic resonance imaging (rs-fMRI) and diffusion tensor imaging (DTI), has enabled the non-invasive exploration of the central mechanisms of neuropathic pain, including changes in brain activity and brain microstructure (Chen et al., 2017; Dai et al., 2020; Park et al., 2022). For example, rs-fMRI metrics such as the amplitude of low-frequency fluctuations (ALFF) and its fractional variant (fALFF) have been widely used in chronic pain research to detect abnormalities in regional brain function (Cao et al., 2017; Zou et al., 2008), while the tract-based spatial statistics (TBSS) method is widely employed to assess white matter integrity (Smith et al., 2006; Hubbard et al., 2018). Despite these advancements, studies using such imaging methods to explore the central mechanisms of stSCS remain limited.

Employing a longitudinal design and multimodal imaging techniques (including rs-fMRI and DTI), this study aims to investigate how a 14-day stSCS modulates brain function and white-matter microstructure in patients with PHN. Specifically, we will assess the effects of stSCS on clinical symptom improvement, analyze changes in brain activity through ALFF and fALFF, and screen whole-brain white matter microstructure using TBSS. We will also investigate the relationship between these brain changes and clinical outcomes. Given PHN’s multidimensional affective burden, we focus on two key white matter tracts: the cingulum bundle, involved in pain processing, and the uncinate fasciculus, linked to emotional processing, particularly anxiety and depression (Apkarian et al., 2011; Lieberman et al., 2014; Bubb et al., 2018; Xu et al., 2023; Tranfa and Pontillo, 2025). We will also explore whether the structural characteristics of these tracts relate to clinical or functional changes.

## 2 Materials and methods

### 2.1 Ethics and participants

This study was approved by the Ethics Committee of the Second Affiliated Hospital of Guangxi Medical University [approval no. 2024-KY(1080)]. All participants provided written informed consent. Between January and August 2025, 20 patients with PHN were recruited.

Inclusion criteria were: (1) age between 40 and 80 years; (2) PHN duration of at least 3 months; (3) insufficient pain relief at least 2 weeks of pregabalin at the maximum tolerated dose; (4) baseline Numeric Rating Scale (NRS) score ≥5; (5) pain confined to unilateral thoracic dermatomes; (6) right-handedness.

Exclusion criteria were: (1) major systemic or central nervous system disorders, except well-controlled hypertension or diabetes; (2) American Society of Anesthesiologists (ASA) classification >II; (3) history of substance abuse; (4) significant neuroimaging abnormalities; (5) previous spinal cord stimulation treatment. After quality control, three participants were excluded due to excessive head motion (mean Frame-wise Displacement [FD] Jenkinson >0.2 mm), leaving 17 for final analysis. Participant demographics are summarized in Table 1.

**Table 1:** Participants demographic and clinical characteristics.

Characteristic	*N* = 17	*p*-value
Age (years)	65.3 ± 9.3	–
Sex		–
Male	13 (76%)	
Female	4 (24%)	
PHN duration (months)	5.0 (4.0, 12.0)	–
Pain laterality		–
Left	9 (53%)	
Right	8 (47%)	
NRS		<0.001
Baseline	6.4 ± 1.0	
After stSCS	2.9 ± 1.2	
PSQI		<0.001
Baseline	14.0(12.0, 17.0)	
After stSCS	9.0(7.0, 10.0)	
HADS-anxiety		<0.001
Baseline	10.6 ± 3.0	
After stSCS	7.0 ± 3.0	
HADS-depression		<0.001
Baseline	12.2 ± 2.3	
After stSCS	8.0 ± 2.1	

Categorical variables are shown as *n* (%); continuous variables as means ± SD or median (IQR) based on normality testing (Shapiro–Wilk); *p*-values from Paired Wilcoxon Signed-Rank Test.

### 2.2 Study design and clinical assessments

This study employed a longitudinal, single-arm, pre-post design. Participants underwent a 14-day continuous stSCS treatment while continuing pregabalin at the maximum tolerated dose as part of a standardized medication regimen. Intolerable breakthrough pain was managed with intramuscular tramadol hydrochloride (50 mg, up to twice daily). During stimulation, 7 of 17 participants required tramadol (1–5 injections) for breakthrough pain. Clinical outcomes, including the Numeric Rating Scale (NRS) for pain intensity, Hospital Anxiety and Depression Scale (HADS) for anxiety (HADS-A) and depression (HADS-D), and Pittsburgh Sleep Quality Index (PSQI) for sleep quality, were assessed at baseline (pre-stSCS) and on day 3 post-stSCS (after the 14-day stimulation period).

### 2.3 stSCS procedure

The stSCS intervention was performed under local anesthesia and C-arm fluoroscopic guidance. An 8-contact stimulation lead (Medtronic 3873 Test Stimulation Lead) was implanted into the epidural space and precisely positioned at the target spinal segment. The final position was confirmed when intraoperative testing achieved >80% paresthesia coverage of the pain area. Following implantation, a 14-day continuous tonic stimulation was delivered using a frequency of 40–60 Hz and a pulse width of 300–500 μs. During the 14-day treatment, stimulation frequency and pulse width were managed by the physician. In contrast, patients were instructed to self-adjust the current amplitude (in 0.1 mA steps) within a safe range (up to 5.0 mA) to maintain ‘optimal paresthesia,’ defined as comfortable stimulation covering >80% of the painful area without discomfort.

### 2.4 MRI data acquisition

All MRI scans were performed on a 3.0 T Siemens MAGNETOM Vida scanner at two time points: pre-stSCS and post-stSCS (day 3 after the 14-day stimulation). Participants were instructed to remain awake with eyes closed and wore an eye mask.

T1-weighted structural images were obtained using a Magnetization Prepared Rapid Gradient Echo (MPRAGE) sequence with parameters: Field of View (FoV) = 256 × 256 mm; matrix size = 256 × 256; Repetition Time (TR) = 2000 ms; Echo Time (TE) = 1.90 ms; Flip Angle (FA) = 8°; isotropic voxel size = 1.0 mm^3^.

Rs-fMRI data were acquired using an Echo Planar Imaging (EPI) sequence with parameters: FoV = 224 × 224 mm; matrix size = 64 × 64; slices = 36; thickness = 3.0 mm; gap = 0.9 mm; TR = 2000 ms; TE = 30 ms; FA = 90°; 240 volumes (480 s); GRAPPA acceleration factor = 2.

DTI data were acquired using a 2D EPI sequence with parameters: FoV = 256 × 256 mm; matrix size = 128 × 128; slices = 66; thickness = 2.0 mm; TR = 7,900 ms; TE = 86 ms; FA = 90°; 30 directions at b = 1,000 and 2000 s/mm^2^, with b = 0 images; GRAPPA employed.

### 2.5 fMRI data processing and analysis

The rs-fMRI data were preprocessed and analyzed using the DPABI toolbox (version V9.0_250415) (Yan et al., 2016). Preprocessing steps included: (1) removal of the first 10 time points; (2) slice-timing correction; (3) realignment; (4) segmentation using New Segment and Diffeomorphic Anatomical Registration Through Exponentiated Lie Algebra (DARTEL); (5) nuisance covariate regression incorporating Friston 24 parameters, head motion scrubbing regressors, and signals from white matter and cerebrospinal fluid; (6) spatial normalization via DARTEL; and (7) detrending. ALFF and fALFF maps were then computed, with the 0.01–0.08 Hz band as the low-frequency component. The maps were spatially smoothed with a 6 mm full-width at half-maximum (FWHM) Gaussian kernel.

Paired *t*-tests were conducted voxel-wise to compare post-stSCS vs. pre-stSCS ALFF and fALFF maps, using a group-level gray matter (GM) mask (mean DARTEL GM segments, threshold >0.2). Head motion was strictly controlled. The mean FD was 0.11 ± 0.04 mm for the pre-stSCS session and 0.09 ± 0.04 mm for the post-stSCS session. No significant difference in head motion was observed between the two time points (*p* = 0.18). Mean FD was included as a covariate. Multiple comparisons were corrected using non-parametric permutation testing (5,000 permutations) with TFCE (*p* < 0.05, family-wise error [FWE]-corrected, two-tailed) (Chen et al., 2018).

### 2.6 DTI data processing and analysis

DTI data were preprocessed using FSL (version 6.0.7.18) (Jenkinson et al., 2012) and MRtrix3 (version 3.0.7-16-g863441d0) (Tournier et al., 2019). The pipeline included: (1) denoising (Veraart et al., 2016); (2) Gibbs artifact removal (Kellner et al., 2016); (3) correction for eddy currents and head motion (Andersson and Sotiropoulos, 2016); and (4) bias field correction. All participant data met quality control standards. The average absolute translation was 0.16 ± 0.06 mm (Max: 1.24 mm) and the average rotation was 0.14 ± 0.06 degrees (Max: 1.98 degrees). No significant differences in motion parameters were found between time points (*p* > 0.05). To generate fractional anisotropy (FA), mean diffusivity (MD), axial diffusivity (AD), and radial diffusivity (RD) maps, the diffusion tensor model was fitted using weighted least squares (dtifit), which was applied to the b = 1,000 s/mm^2^ shell and the b = 0 images.

Whole-brain white matter changes were analyzed using TBSS, according to the standard pipeline (Smith et al., 2006), which includes non-linear registration to a standard template and projection onto a mean FA skeleton (thresholded at FA > 0.2). A paired *t*-test was subsequently performed using FSL’s randomize (5,000 permutations; TFCE) to assess significant longitudinal changes (two-tailed) in white matter integrity following stSCS.

For *a priori* regions of interest (ROIs), masks for the cingulum bundle (JHU indices 4 and 5) and uncinate fasciculus (JHU indices 16 and 17) were obtained from the JHU-ICBM-tracts-maxprob-thr0-1 mm atlas (Hua et al., 2008) and intersected with the mean FA skeleton (FA > 0.2). Mean values of FA, MD, AD, and RD were extracted for each ROI. These extracted metrics were used both to assess longitudinal changes (FDR-corrected paired *t*-tests) and for exploratory correlation analyses (using the baseline values).

### 2.7 Correlation analyses

All statistical analyses were performed using R (version 4.3.1) (R Core Team, 2023). Given the small sample size, non-parametric Spearman rank correlations were used. To represent clinical improvement, *Δ* clinical values were calculated as pre-stSCS—post-stSCS for the four clinical metrics (NRS, HADS-A, HADS-D, PSQI). For functional changes, Δ values for fALFF were computed as post-stSCS—pre-stSCS from significant clusters identified. These ΔfALFF values were correlated with the Δ clinical values; *p*-values were FDR-corrected across all tests.

As exploratory analyses to investigate potential predictors of treatment response, the 8 baseline DTI ROI metrics were correlated with the clinical change scores and the Δ functional values. *p*-values were FDR-corrected separately for each set. Significant findings were further assessed with partial correlations, controlling for age, sex, and disease duration.

## 3 Results

### 3.1 Clinical outcomes

**Figure 1:**
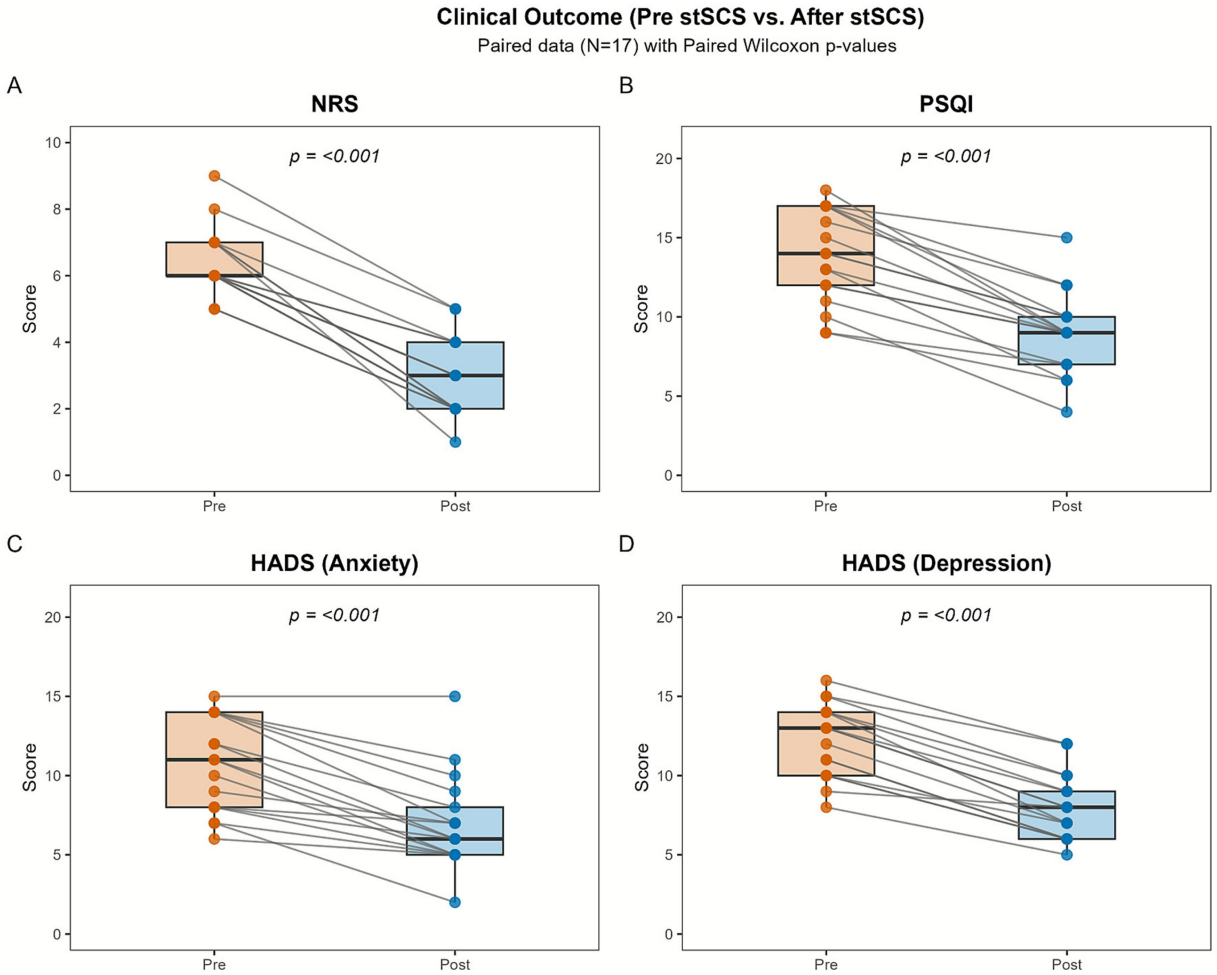
Longitudinal changes in clinical scores for all participants (*N* = 17). Comparisons of **(A)** NRS, **(B)** PSQI, **(C)** HADS-anxiety, and **(D)** HADS-depression scores at pre-stSCS and post-stSCS. Gray lines connect data from individual participants. Boxplots illustrate the median and interquartile range (IQR). *p*-Values derived from paired Wilcoxon Signed-Rank Test.

Following the 14-day stSCS intervention, statistically significant improvements were observed in all clinical outcomes (Table 1; Figure 1). Wilcoxon signed-rank tests revealed significant reductions in post-stSCS scores compared to baseline for NRS (*Z* = −3.671, *p* < 0.001), PSQI (*Z* = −3.635, *p* < 0.001), HADS-Anxiety (*Z* = −3.608, *p* < 0.001), and HADS-Depression (*Z* = −3.663, *p* < 0.001).

### 3.2 fMRI results

The longitudinal voxel-wise fMRI analysis (TFCE, *p* < 0.05, FWE-corrected) showed that ALFF maps yielded no significant clusters following stSCS.

**Figure 2:**
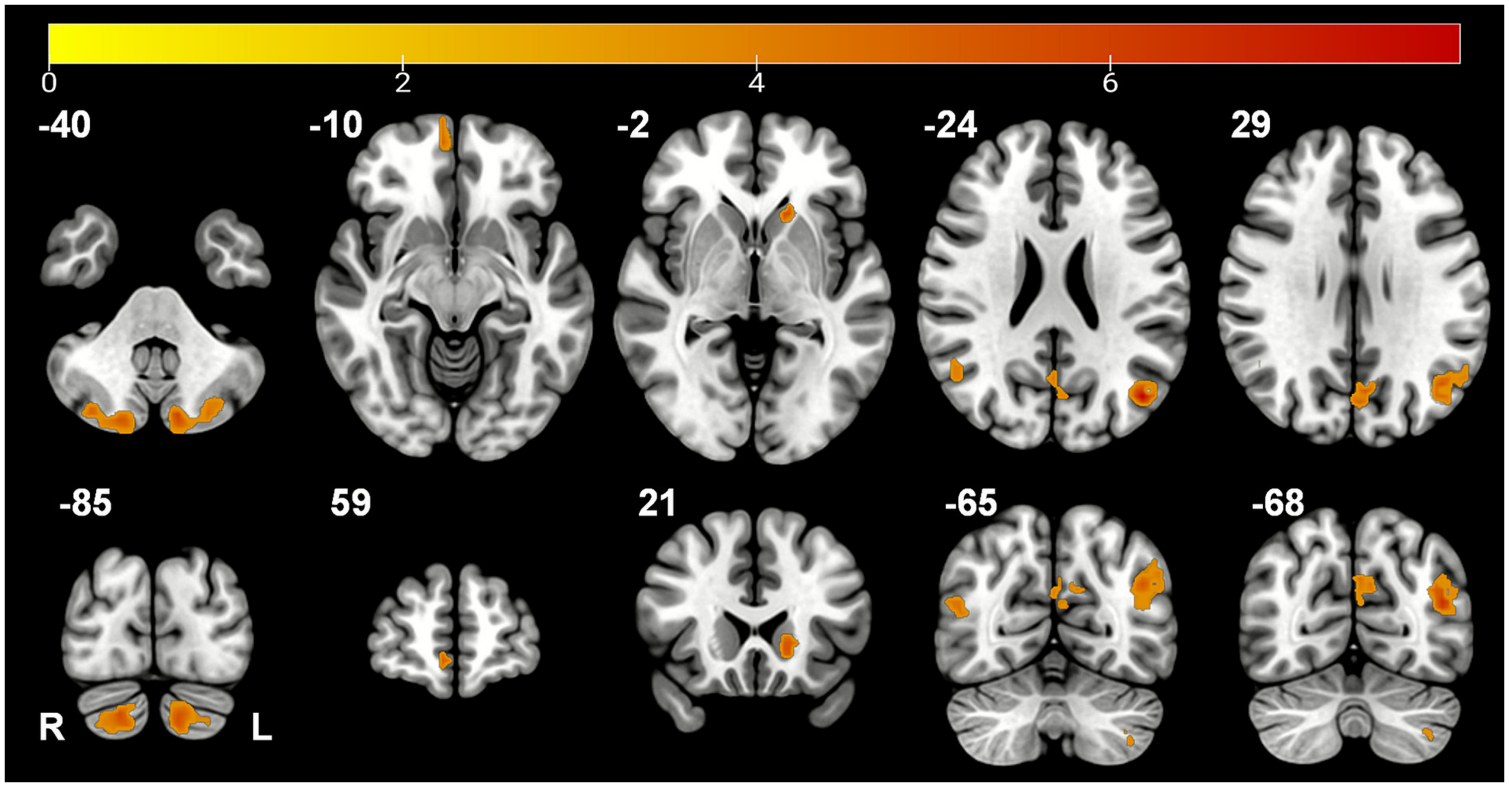
Brain regions with significant fALFF increases following stSCS. Results are overlaid on a standard MNI template, shown in axial (top row) and coronal (bottom row) views. Numbers indicate the respective MNI slice coordinate. All clusters are corrected (TFCE, *p* < 0.05 FWE) and thresholded at *k* ≥ 15 voxels. The color bar indicates *T*-statistic values. R = right.

In contrast, the same analysis of fALFF maps revealed 12 clusters with significant increases (Supplementary Table S1). To enhance robustness, the seven primary clusters (k ≥ 15 voxels), which include regions like the Cerebellum Crus II, Angular Gyrus, and Caudate Nucleus (Table 2; Figure 2), were retained for subsequent correlation analyses.

**Table 2:** Brain regions with increased fALFF following stSCS (TFCE, k ≥ 15).

Cluster	Brain region	Hemi	*k* (voxels)	Peak MNI (x, y, z)	Peak intensity
C1	Cerebellum Crus II	L	118	−12, −81, −39	6.250
C2	Angular gyrus/Middle temporal gyrus	L	114	−45, −72, 24	8.408
C3	Cerebellum Crus II	R	106	18, −84, −36	5.782
C4	Cuneus/Precuneus	L	49	−6, −72, 30	5.782
C5	Middle temporal gyrus/Angular gyrus	R	42	54, −60, 12	6.066
C6	Medial orbitofrontal gyrus	R	18	6, 57, −9	5.391
C7	Caudate nucleus/Putamen	L	15	−18, 24, 0	5.746

Results from paired *t*-test (post vs. pre), corrected using permutation test (TFCE, *p* < 0.05 FWE).

### 3.3 DTI results

In contrast to the fALFF changes, the 14-day stSCS intervention did not induce any detectable structural changes. The whole-brain TBSS analysis (testing two-tailed) found no significant changes on the white matter skeleton for FA, MD, AD, or RD (all *p* > 0.05, FWE-corrected). Similarly, the ROI-based longitudinal analysis of the cingulum and uncinate fasciculus also showed no significant differences in any of the 8 DTI metrics after FDR correction (all *p*.adj > 0.8). Specifically, for the cingulum bundle, the mean FA was 0.52 ± 0.02 at baseline and 0.52 ± 0.02 post-treatment; MD was 0.77 ± 0.03 × 10^−3^ mm^2^/s at baseline and 0.77 ± 0.03 × 10^−3^ mm^2^/s post-treatment. The uncinate fasciculus showed similarly stable microstructural metrics (FA: 0.43 ± 0.03 vs. 0.43 ± 0.03; MD: 0.79 ± 0.04 vs. 0.79 ± 0.04 × 10^−3^ mm^2^/s), confirming the absence of rapid structural plasticity. Detailed results for both TBSS and ROI analyses are presented in Supplementary Tables S2 and S3, respectively.

### 3.4 Brain-clinical correlations analyses

We next examined the relationship between fALFF and clinical improvement, using the seven primary fALFF clusters. This analysis revealed one significant correlation that survived FDR correction (detailed in Supplementary Table S4). We found that fALFF changes in the right medial orbitofrontal gyrus (C6) were significantly correlated with reductions in NRS scores (Spearman’s *r* = 0.71, *p*.adj = 0.0364). This finding remained robust in a post-hoc partial correlation analysis controlling for age and disease duration (partial Spearman’s *r* = 0.703, *p* = 0.00345, uncorrected).

**Figure 3:**
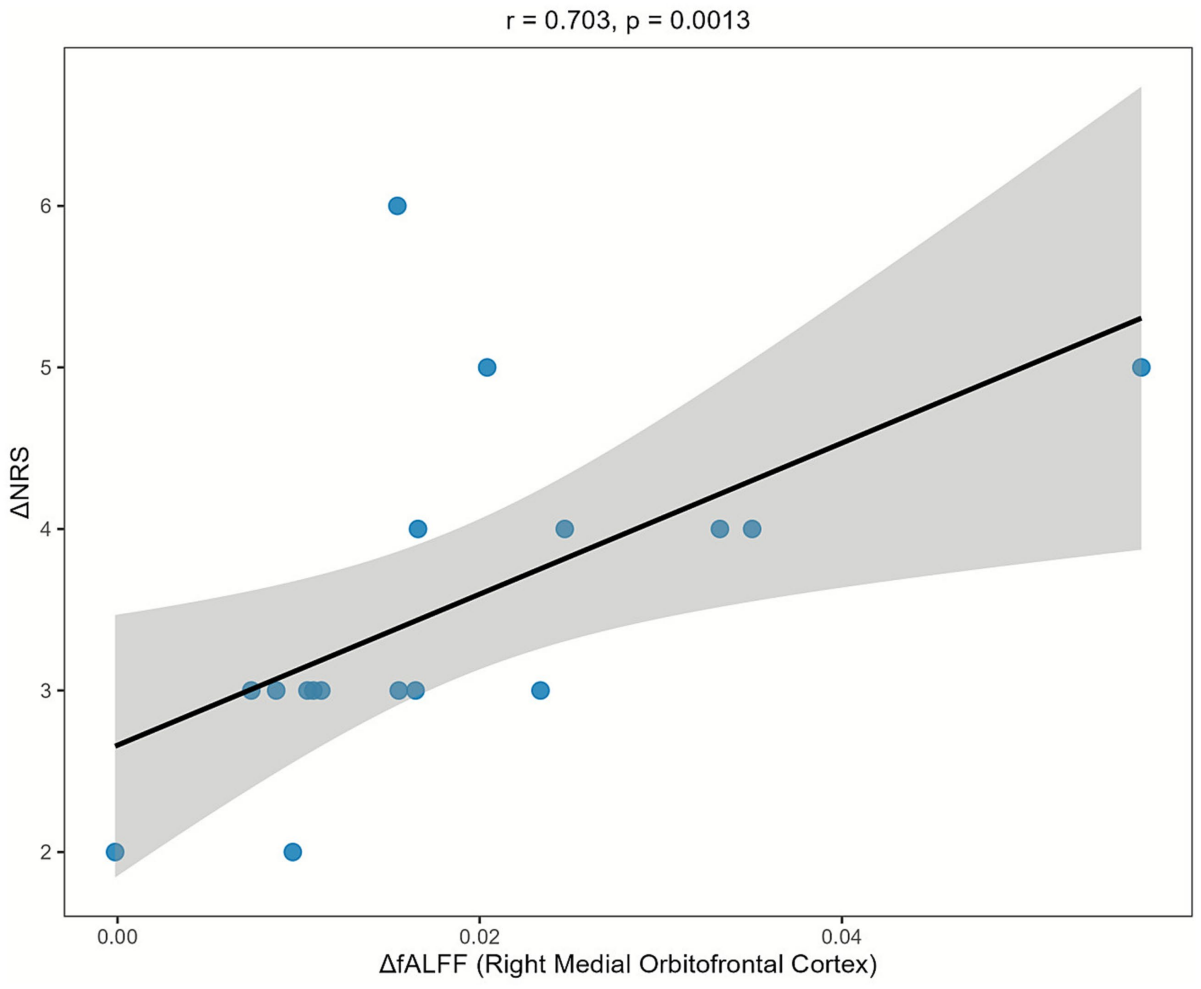
Correlation between fALFF changes in the right medial orbitofrontal cortex (C6) and pain improvement. Scatter plot (*N* = 17) showing the relationship between ΔfALFF (C6, Right Medial Orbitofrontal Gyrus) and ΔNRS. The line indicates the linear regression fit with 95% confidence interval. Statistics: Spearman’s *r* = 0.71, uncorrected *p* = 0.0013.

No other fALFF clusters showed FDR-corrected correlations with any clinical metrics (all *p*.adj > 0.05). The significant correlation is visualized in Figure 3.

### 3.5 Exploratory baseline DTI correlations

Given the absence of longitudinal structural changes, we proceeded to explore whether baseline (Pre-stSCS) DTI metrics from the *a priori* ROIs (cingulum and uncinate fasciculus) correlated with clinical or functional changes. In the DTI-to-Clinical analysis (Supplementary Table S5), no significant correlations were found between these baseline ROI metrics and any subsequent clinical improvements (all *p*.adj > 0.5).

**Figure 4:**
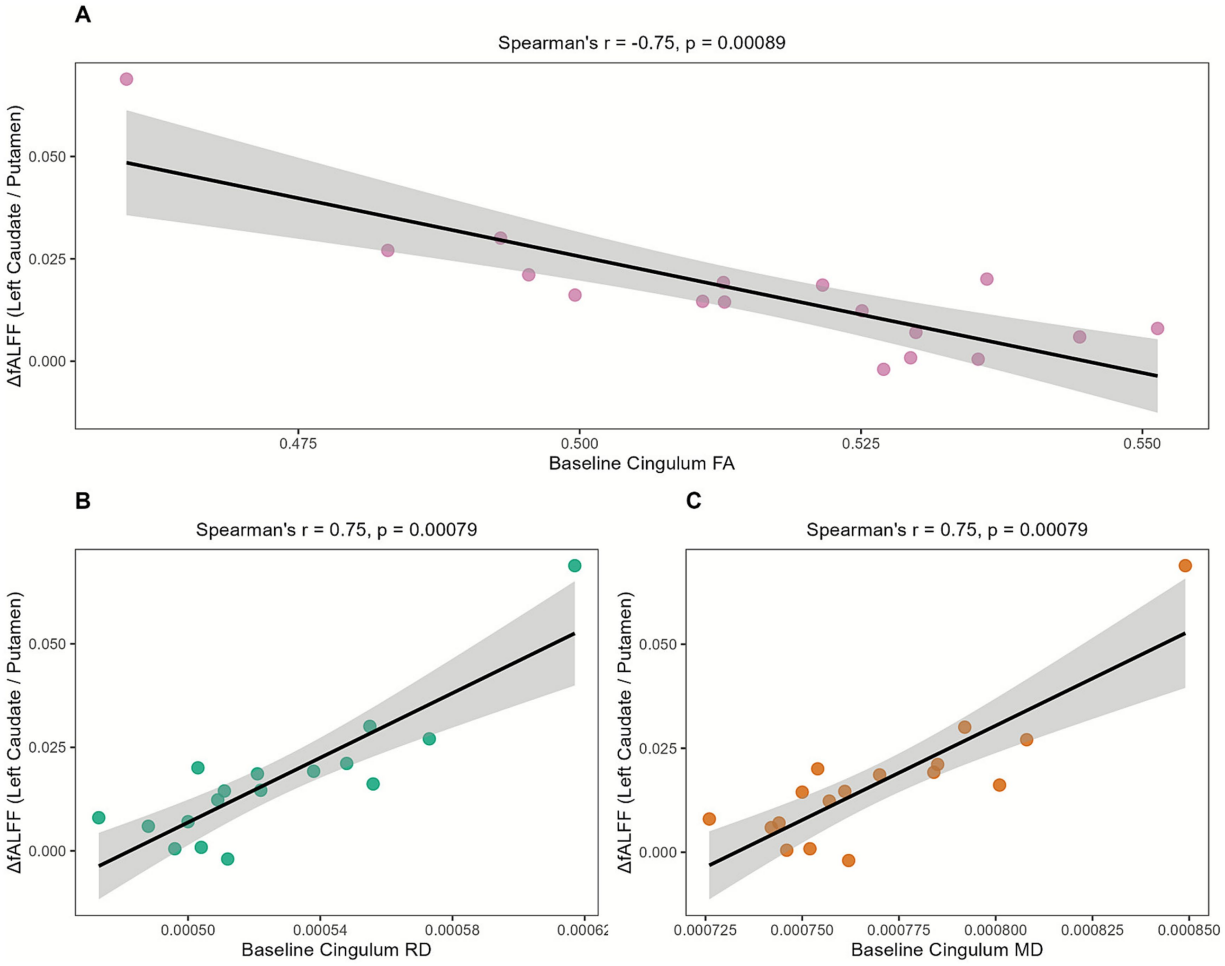
Relationship between baseline DTI metrics in the cingulum bundle and fALFF changes in the left caudate/putamen (C7). Scatter plots (*N* = 17) showing the relationship between ΔfALFF (C7, the left caudate/putamen) and **(A)** baseline cingulum FA, **(B)** baseline cingulum RD, and **(C)** baseline cingulum MD. The line indicates the linear regression fit with 95% confidence interval. Statistics shown are Spearman’s *r* and uncorrected *p*-values.

However, the DTI-to-Function analysis (Supplementary Table S6) revealed significant correlations. The baseline microstructural integrity of the cingulum significantly correlated with fALFF changes in the left caudate/putamen (C7). Specifically, baseline cingulum FA showed a strong negative correlation (Spearman’s *r* = −0.75, *p*.adj = 0.017), while both MD (*r* = 0.75, *p*.adj = 0.0167) and RD (*r* = 0.75, *p*.adj = 0.0167) showed strong positive correlations. All three correlations remained robust in post-hoc partial correlation analyses controlling for age and disease duration (all uncorrected *p* < 0.013). These results are detailed in Figure 4 and Supplementary Table S6.

## 4 Discussion

This study utilized a longitudinal, multimodal neuroimaging design to investigate the potential effects of a 14-day stSCS treatment on clinical symptoms and central neural mechanisms in patients with PHN. We observed that following stSCS treatment, patients achieved significant clinical improvements in pain intensity (NRS), anxiety and depression (HADS), and sleep quality (PSQI). On the neuroimaging level, we found that stSCS induced a reorganization of local brain function, manifested as significant increases in fALFF values in several brain regions; however, no detectable changes in white matter microstructure were observed during this period. Further correlation analyses revealed potential links between brain-clinical and structure–function relationships. Specifically, fALFF changes in the medial orbitofrontal cortex (C6) were positively correlated with the degree of pain improvement. Furthermore, we found that baseline white matter integrity in the cingulum was significantly correlated with treatment-induced fALFF changes in the caudate/putamen area (C7).

Our observation of significant improvements across pain, mood, and sleep metrics is consistent with a growing body of evidence supporting the multimodal efficacy of stSCS for PHN (Li et al., 2025; Sheng et al., 2022; Zuo et al., 2024). Recent meta-analyses confirm that stSCS provides effective short-term relief for pain and associated comorbidities in PHN, often superior to other interventional techniques (Liu et al., 2025). This clinical improvement was paralleled by a reorganization of local brain function. Both ALFF and fALFF are metrics designed to quantify the intensity of spontaneous, low-frequency neural activity, which is considered a proxy for intrinsic brain function (Zou et al., 2008; Zang et al., 2007). However, fALFF is a normalized ratio of this low-frequency power to the power across the entire frequency spectrum. Consequently, fALFF is considered less susceptible to physiological noise (such as cardiac and respiratory artifacts) than the absolute measure of ALFF and thus may have greater sensitivity for detecting longitudinal changes (Zou et al., 2008). This methodological distinction may explain why our ALFF analysis yielded no significant clusters, while fALFF revealed robust changes. Our observation of increased fALFF in the dorsal striatum (caudate/putamen, C7) is noteworthy. The striatum has been previously implicated in PHN pathophysiology (Tang et al., 2021). Our finding of increased fALFF in this region post-treatment is consistent with other longitudinal studies, which have also reported increased fALFF in the bilateral caudate of PHN patients 6 months after successful pain treatment (Zhang et al., 2020). The changes observed in other large clusters, such as the cerebellum (C1/C3), angular gyrus (C2/C5), and precuneus (C4), are also in line with literature identifying these areas as part of the extended brain network affected by PHN (Chen et al., 2017; Huang et al., 2020). This modulation of parietal networks is also broadly consistent with another recent stSCS study, which reported functional changes in the precuneus and inferior parietal lobule using different metrics (Fan et al., 2022).

Conversely, we did not detect significant longitudinal changes in white matter microstructure after stSCS. This finding is plausible and consistent with the known timelines of neural plasticity, as plasticity in white matter structure is broadly understood to occur over a much longer timescale than functional reorganization. For instance, Zhang et al. observed structural normalization in PHN patients only at a 6-month follow-up (Zhang et al., 2020). Similarly, longitudinal studies in other chronic pain populations, such as osteoarthritis, have found that structural brain changes—encompassing both gray matter volume and white matter integrity—were observed several months after successful joint replacement surgery (Lewis et al., 2018). Taken together, our findings suggest that the rapid clinical efficacy of stSCS is primarily mediated by fast-acting functional reorganization, as indexed by our fALFF results, rather than by immediate structural alterations in microstructure.

Having established the group-level functional changes, we next sought to identify the neural correlates of the clinical improvement. Although the overall treatment effect was significant, we noted heterogeneity in the clinical response: 12 of 17 (70.6%) participants achieved a clinically significant pain reduction (50%), while 5 did not. This observed variance in treatment response led us to explore whether these individual differences were associated with the observed functional brain changes. Our analysis revealed a specific positive correlation between the magnitude of pain reduction (∆NRS) and fALFF increases in the right medial orbitofrontal cortex (mOFC, C6). The mOFC is critically involved in the cognitive evaluation of pain; specifically, it assesses the affective value and context of a nociceptive stimulus, rather than its raw sensory intensity (Becker et al., 2017; Winston et al., 2014).

Our finding that the OFC is a key target of stSCS is consistent with recent literature. For instance, a recent stSCS study with a similar design also identified the OFC as a primary site of modulation (Bu et al., 2023). However, while their study found a decrease in dynamic ALFF (dALFF) correlated with sleep improvement, our study observed an increase in static fALFF correlated with pain relief. This discrepancy likely stems from the use of different metrics, which capture distinct aspects of neural activity—our finding may reflect normalization of baseline hypoactivity, while theirs may indicate stabilization of erratic activity. Nonetheless, both studies highlight the OFC as a key treatment site. Activity in the mOFC has been associated with analgesia induced by reward and positive expectation. It is hypothesized that this process mediates pain reduction by changing the significance or value of the pain signal, as opposed to directly blocking the nociceptive input itself (Becker et al., 2017). It is therefore plausible that our result reflects stSCS modulating the activity of the mOFC, which in turn may enhance the cognitive capacity to reappraise and inhibit pain.

Our exploratory analysis also revealed a significant relationship between baseline structural integrity and subsequent functional change. DTI metrics provide biological insights into white matter microstructure: FA reflects the directional coherence of water diffusion, with lower values indicating poorer tract organization, while Mean MD and RD measure the overall magnitude of diffusion. We found that reduced baseline integrity in the cingulum bundle (evidenced by lower FA and higher MD/RD) was strongly correlated with greater fALFF increases in the dorsal striatum (C7, caudate/putamen). This finding links two regions highly relevant to PHN pathophysiology. First, the dorsal striatum (including the caudate and putamen) has been identified in studies as a site of functional abnormality in PHN (Yang et al., 2023; Liu et al., 2013), with activity in the caudate showing reversal following long-term pain relief, suggesting its potential as a treatment target (Zhang et al., 2020). Second, the cingulum bundle, a major white matter tract connecting frontal and limbic regions, has been shown to exhibit significant microstructural alterations in PHN patients, which correlate with emotional changes and disease duration (Wu et al., 2022).

Our study provides preliminary evidence linking these two observations. While the mechanism requires further investigation, this structure–function relationship suggests a potential compensatory process. The cingulum bundle provides critical anatomical connectivity between affective-motivational circuits (like the anterior cingulate cortex) and the dorsal striatum. It is plausible that individuals with greater pre-existing structural disruption in this pathway may possess a greater capacity for striatal functional plasticity in response to stSCS. This finding, though requiring validation in larger cohorts, suggests that baseline DTI metrics could be explored as a potential biomarker for predicting functional responsiveness to neuromodulation.

Several limitations of this study should be noted. First, the most significant limitation is the single-arm, pre-post design, which lacks a sham-control group. Therefore, we cannot completely attribute the observed clinical and functional changes solely to stSCS, as placebo effects or the natural history of the condition cannot be ruled out. Future studies utilizing a randomized controlled trial (RCT) design are needed to definitively validate these findings. Second, the sample size (*N* = 17) was modest and derived from a single center. Therefore, our correlation analyses, particularly the exploratory structure–function findings, should be interpreted as preliminary and require validation in larger cohorts. Third, the 14-day follow-up period, while appropriate for assessing rapid functional changes, does not allow for conclusions about the long-term durability of these effects. Finally, the concurrent medication regimen represents a potential confounding factor, as pregabalin and tramadol are reported to influence mood and sleep quality (Bumpus, 2020; Hong et al., 2022). However, pregabalin dosage in our cohort was highly standardized, and post-hoc sensitivity analyses revealed no significant differences in clinical or functional outcomes between patients requiring rescue tramadol and those who did not (all *p* > 0.05; individual data in Supplementary Table S7). This suggests that the observed effects are primarily driven by stSCS, although potential subtle influences cannot be entirely ruled out.

## 5 Conclusion

In conclusion, our findings indicate that a 14-day stSCS intervention significantly alleviates pain, mood disturbances, and sleep issues in PHN patients. Multimodal imaging further suggests that these benefits stem from rapid functional brain reorganization, rather than immediate white matter microstructural changes. This reorganization is underscored by two preliminary findings: fALFF increases in the right medial orbitofrontal cortex correlated with pain relief, and baseline cingulum microstructural integrity predicted fALFF changes in the dorsal striatum. Collectively, these results offer initial mechanistic insights into stSCS. Preliminary evidence suggests that baseline brain structure may serve as a potential biomarker for treatment response, warranting validation in larger cohorts.

## Data Availability

The original contributions presented in the study are included in the article/Supplementary material, further inquiries can be directed to the corresponding authors.
