# Supplementary material for: Central effects of short-term spinal cord stimulation in postherpetic neuralgia: a longitudinal fMRI and DTI study
Source: Front Neurosci. 2026 Jan 13;19:1744783. doi: 10.3389/fnins.2025.1744783 (PMC12835296; doi:10.3389/fnins.2025.1744783)
Supplement: Supplementary file 1 [file Table_1.docx]

| **Cluster** | **Brain Region** | **Hemi** | **k(voxels)** | **Peak MNI**  **(x, y, z)** | **Peak Intensity** |
| --- | --- | --- | --- | --- | --- |
| C1 | Cerebellum Crus II | L | 118 | -12, -81, -39 | 6.250 |
| C2 | Angular Gyrus / Middle Temporal Gyrus | L | 114 | -45, -72, 24 | 8.408 |
| C3 | Cerebellum Crus II | R | 106 | 18, -84, -36 | 5.782 |
| C4 | Cuneus / Precuneus | L | 49 | -6, -72, 30 | 5.782 |
| C5 | Middle Temporal Gyrus / Angular Gyrus | R | 42 | 54, -60, 12 | 6.066 |
| C6 | Medial Orbitofrontal Gyrus | R | 18 | 6, 57, -9 | 5.391 |
| C7 | Caudate Nucleus / Putamen | L | 15 | -18, 24, 0 | 5.746 |
| C8 | Superior Medial Frontal Gyrus | R | 5 | 15, 51, 30 | 4.006 |
| C9 | Supramarginal Gyrus | L | 3 | -63, -27, 24 | 5.070 |
| C10 | Superior Frontal Gyrus (Area 2) | R | 2 | 21, 42, 30 | 4.114 |
| C11 | Middle Frontal Gyrus (Area 2) | L | 1 | -36, 45, -9 | 5.424 |
| C12 | Posterior Cingulate Gyrus | L | 1 | -12, -51, 27 | 5.054 |

**Supplementary Table S1.** All brain regions showing significant fALFF increases following stSCS

Results from paired t-test (Post vs. Pre), corrected using Permutation Test (TFCE, *p* < 0.05 FWE). Abbreviations: fALFF, fractional amplitude of low-frequency fluctuation; Hemi, Hemisphere; k, cluster size (voxels); MNI, Montreal Neurological Institute.
