# Supplementary material for: Central effects of short-term spinal cord stimulation in postherpetic neuralgia: a longitudinal fMRI and DTI study
Source: Front Neurosci. 2026 Jan 13;19:1744783. doi: 10.3389/fnins.2025.1744783 (PMC12835296; doi:10.3389/fnins.2025.1744783)
Supplement: Supplementary file 2 [file Table_2.DOCX]

**Supplementary Table S2.** Whole-brain TBSS results for longitudinal changes (N=17).

| **Contrast** | **Metric** | ***p*-value (FWE-corrected)** |
| --- | --- | --- |
| Post vs. Pre | FA | 0.066 |
|  | MD | 0.926 |
|  | RD | 0.914 |
|  | AD | 0.777 |
| Pre vs. Post | FA | 0.955 |
|  | MD | 0.239 |
|  | RD | 0.094 |
|  | AD | 0.331 |

*p*-values derived from permutation testing with TFCE (FWE-corrected). Abbreviations: TBSS, Tract-Based Spatial Statistics; FWE, Family-Wise Error; FA, Fractional Anisotropy; MD, Mean Diffusivity; AD, Axial Diffusivity; RD, Radial Diffusivity.
