# Supplementary material for: Central effects of short-term spinal cord stimulation in postherpetic neuralgia: a longitudinal fMRI and DTI study
Source: Front Neurosci. 2026 Jan 13;19:1744783. doi: 10.3389/fnins.2025.1744783 (PMC12835296; doi:10.3389/fnins.2025.1744783)
Supplement: Supplementary file 3 [file Table_3.DOCX]

**Supplementary Table S3.** ROI-based longitudinal changes in DTI metrics (N=17).

| **Tract** | **Metric** | **Pre-stSCS**  **(Mean ± SD)** | **Post-stSCS**  **(Mean ± SD)** | ***p*-value (uncorrected)** | ***p*-value (FDR-corrected)** |
| --- | --- | --- | --- | --- | --- |
| **Cingulum** | FA | 0.52 ± 0.02 | 0.52 ± 0.02 | 0.306 | 0.826 |
|  | MD | 0.77 ± 0.03 | 0.77 ± 0.03 | 0.722 | 0.826 |
|  | AD | 1.26 ± 0.02 | 1.26 ± 0.03 | 0.943 | 0.943 |
|  | RD | 0.53 ± 0.04 | 0.52 ± 0.04 | 0.552 | 0.826 |
| **Uncinate** | FA | 0.43 ± 0.03 | 0.43 ± 0.03 | 0.329 | 0.826 |
|  | MD | 0.79 ± 0.04 | 0.79 ± 0.04 | 0.670 | 0.826 |
|  | AD | 1.18 ± 0.03 | 1.17 ± 0.03 | 0.587 | 0.826 |
|  | RD | 0.60 ± 0.04 | 0.59 ± 0.04 | 0.469 | 0.826 |

Values for MD, AD, and RD are presented in ×10−3 mm2/s. FA is dimensionless. *p*-values derived from paired t-tests (post vs. pre) and subsequently FDR-corrected. Abbreviations: ROI, Region of Interest; FA, Fractional Anisotropy; MD, Mean Diffusivity; AD, Axial Diffusivity; RD, Radial Diffusivity.
