# Supplementary material for: Central effects of short-term spinal cord stimulation in postherpetic neuralgia: a longitudinal fMRI and DTI study
Source: Front Neurosci. 2026 Jan 13;19:1744783. doi: 10.3389/fnins.2025.1744783 (PMC12835296; doi:10.3389/fnins.2025.1744783)
Supplement: Supplementary file 4 [file Table_4.DOCX]

**Supplementary Table S4.** Correlation analysis between fALFF changes and clinical improvements (N=17).

| **fALFF Cluster** | **Clinical Metric** | **Spearman's r** | **p-value (uncorrected)** | **p-value (FDR-corrected)** |
| --- | --- | --- | --- | --- |
| Delta_fALFF_C1_L_Cereb | Delta_NRS | 0.057 | 0.827 | 0.981 |
| Delta_fALFF_C1_L_Cereb | Delta_HADS_A | -0.038 | 0.884 | 0.981 |
| Delta_fALFF_C1_L_Cereb | Delta_HADS_D | -0.210 | 0.427 | 0.892 |
| Delta_fALFF_C1_L_Cereb | Delta_PSQI | 0.130 | 0.607 | 0.960 |
| Delta_fALFF_C2_L_Angular | Delta_NRS | -0.017 | 0.948 | 0.981 |
| Delta_fALFF_C2_L_Angular | Delta_HADS_A | -0.150 | 0.572 | 0.960 |
| Delta_fALFF_C2_L_Angular | Delta_HADS_D | 0.150 | 0.553 | 0.960 |
| Delta_fALFF_C2_L_Angular | Delta_PSQI | 0.410 | 0.106 | 0.742 |
| Delta_fALFF_C3_R_Cereb | Delta_NRS | -0.009 | 0.972 | 0.981 |
| Delta_fALFF_C3_R_Cereb | Delta_HADS_A | -0.240 | 0.360 | 0.892 |
| Delta_fALFF_C3_R_Cereb | Delta_HADS_D | 0.088 | 0.738 | 0.981 |
| Delta_fALFF_C3_R_Cereb | Delta_PSQI | 0.210 | 0.411 | 0.892 |
| Delta_fALFF_C4_L_Precun | Delta_NRS | -0.061 | 0.815 | 0.981 |
| Delta_fALFF_C4_L_Precun | Delta_HADS_A | 0.019 | 0.944 | 0.981 |
| Delta_fALFF_C4_L_Precun | Delta_HADS_D | 0.290 | 0.252 | 0.882 |
| Delta_fALFF_C4_L_Precun | Delta_PSQI | 0.600 | 0.0112 | 0.157 |
| Delta_fALFF_C5_R_Temporal | Delta_NRS | 0.340 | 0.176 | 0.882 |
| Delta_fALFF_C5_R_Temporal | Delta_HADS_A | -0.006 | 0.981 | 0.981 |
| Delta_fALFF_C5_R_Temporal | Delta_HADS_D | 0.300 | 0.243 | 0.882 |
| Delta_fALFF_C5_R_Temporal | Delta_PSQI | 0.130 | 0.617 | 0.960 |
| **Delta_fALFF_C6_R_Frontal** | **Delta_NRS** | **0.710** | **0.0013*** | **0.0364*** |
| Delta_fALFF_C6_R_Frontal | Delta_HADS_A | 0.500 | 0.0424 | 0.396 |
| Delta_fALFF_C6_R_Frontal | Delta_HADS_D | 0.260 | 0.307 | 0.892 |
| Delta_fALFF_C6_R_Frontal | Delta_PSQI | -0.019 | 0.943 | 0.981 |
| Delta_fALFF_C7_L_Caudate | Delta_NRS | 0.230 | 0.384 | 0.892 |
| Delta_fALFF_C7_L_Caudate | Delta_HADS_A | 0.310 | 0.220 | 0.882 |
| Delta_fALFF_C7_L_Caudate | Delta_HADS_D | -0.026 | 0.922 | 0.981 |
| Delta_fALFF_C7_L_Caudate | Delta_PSQI | -0.200 | 0.446 | 0.892 |

Spearman's rank correlations (N=17). p-values (FDR-corrected) were adjusted across all 28 comparisons. * = Significant .Abbreviations: fALFF, fractional amplitude of low-frequency fluctuation; FDR, False Discovery Rate; ns, not significant; L, Left; R, Right; NRS, Numeric Rating Scale; HADS, Hospital Anxiety and Depression Scale; PSQI, Pittsburgh Sleep Quality Index.
