# Supplementary material for: Central effects of short-term spinal cord stimulation in postherpetic neuralgia: a longitudinal fMRI and DTI study
Source: Front Neurosci. 2026 Jan 13;19:1744783. doi: 10.3389/fnins.2025.1744783 (PMC12835296; doi:10.3389/fnins.2025.1744783)
Supplement: Supplementary file 5 [file Table_5.docx]

**Supplementary Table S5.** Exploratory correlation analysis between baseline DTI metrics and clinical improvements (N=17).

| **Baseline DTI Metric (8)** | **Clinical Metric (4)** | **Spearman's r** | **p-value (uncorrected)** | **p-value (FDR-corrected)** |
| --- | --- | --- | --- | --- |
| **pre_FA_Cingulum** | Delta_NRS | -0.400 | 0.109 | 0.580 |
|  | Delta_HADS_A | -0.280 | 0.281 | 0.580 |
|  | Delta_HADS_D | -0.280 | 0.269 | 0.580 |
|  | Delta_PSQI | -0.008 | 0.977 | 0.977 |
| **pre_FA_Uncinate** | Delta_NRS | -0.430 | 0.0888 | 0.580 |
|  | Delta_HADS_A | -0.270 | 0.290 | 0.580 |
|  | Delta_HADS_D | -0.360 | 0.154 | 0.580 |
|  | Delta_PSQI | -0.062 | 0.812 | 0.928 |
| **pre_MD_Cingulum** | Delta_NRS | 0.200 | 0.452 | 0.761 |
|  | Delta_HADS_A | 0.170 | 0.518 | 0.829 |
|  | Delta_HADS_D | 0.280 | 0.279 | 0.580 |
|  | Delta_PSQI | 0.067 | 0.797 | 0.928 |
| **pre_MD_Uncinate** | Delta_NRS | 0.400 | 0.112 | 0.580 |
|  | Delta_HADS_A | 0.300 | 0.234 | 0.580 |
|  | Delta_HADS_D | 0.420 | 0.0905 | 0.580 |
|  | Delta_PSQI | 0.041 | 0.875 | 0.966 |
| **pre_RD_Cingulum** | Delta_NRS | 0.350 | 0.174 | 0.580 |
|  | Delta_HADS_A | 0.260 | 0.311 | 0.585 |
|  | Delta_HADS_D | 0.310 | 0.226 | 0.580 |
|  | Delta_PSQI | 0.072 | 0.783 | 0.928 |
| **pre_RD_Uncinate** | Delta_NRS | 0.370 | 0.142 | 0.580 |
|  | Delta_HADS_A | 0.310 | 0.224 | 0.580 |
|  | Delta_HADS_D | 0.410 | 0.0995 | 0.580 |
|  | Delta_PSQI | 0.015 | 0.955 | 0.977 |
| **pre_AD_Cingulum** | Delta_NRS | -0.029 | 0.913 | 0.974 |
|  | Delta_HADS_A | -0.083 | 0.751 | 0.928 |
|  | Delta_HADS_D | 0.210 | 0.415 | 0.738 |
|  | Delta_PSQI | 0.140 | 0.593 | 0.904 |
| **pre_AD_Uncinate** | Delta_NRS | 0.100 | 0.690 | 0.928 |
|  | Delta_HADS_A | 0.110 | 0.685 | 0.928 |
|  | Delta_HADS_D | 0.460 | 0.0634 | 0.580 |
|  | Delta_PSQI | 0.074 | 0.779 | 0.928 |

Spearman's rank correlations (N=17). *p*-values (FDR-corrected) were adjusted across all 32 comparisons (8 DTI metrics × 4 clinical metrics). Abbreviations: DTI, Diffusion Tensor Imaging; FDR, False Discovery Rate; FA, Fractional Anisotropy; MD, Mean Diffusivity; RD, Radial Diffusivity; AD, Axial Diffusivity; NRS, Numeric Rating Scale; HADS, Hospital Anxiety and Depression Scale; PSQI, Pittsburgh Sleep Quality Index.
