# Supplementary material for: Central effects of short-term spinal cord stimulation in postherpetic neuralgia: a longitudinal fMRI and DTI study
Source: Front Neurosci. 2026 Jan 13;19:1744783. doi: 10.3389/fnins.2025.1744783 (PMC12835296; doi:10.3389/fnins.2025.1744783)
Supplement: Supplementary file 6 [file Table_6.DOCX]

**Supplementary Table S6.** Exploratory correlation analysis between baseline DTI metrics and fALFF changes (N=17).

| **Baseline DTI Metric (8)** | **fALFF Cluster (7)** | **Spearman's r** | **p-value (uncorrected)** | **p-value (FDR-corrected)** |
| --- | --- | --- | --- | --- |
| **pre_FA_Cingulum** | Delta_fALFF_C1_L_Cereb | -0.150 | 0.566 | 0.967 |
|  | Delta_fALFF_C2_L_Angular | 0.034 | 0.898 | 0.967 |
|  | Delta_fALFF_C3_R_Cereb | -0.400 | 0.111 | 0.414 |
|  | Delta_fALFF_C4_L_Precun | -0.003 | 0.996 | 0.996 |
|  | Delta_fALFF_C5_R_Temporal | 0.120 | 0.639 | 0.967 |
|  | Delta_fALFF_C6_R_Frontal | -0.440 | 0.0816 | 0.352 |
|  | **Delta_fALFF_C7_L_Caudate** | **-0.750** | **0.00089** | **0.0167*** |
| **pre_FA_Uncinate** | Delta_fALFF_C1_L_Cereb | -0.044 | 0.869 | 0.967 |
|  | Delta_fALFF_C2_L_Angular | 0.017 | 0.951 | 0.986 |
|  | Delta_fALFF_C3_R_Cereb | -0.230 | 0.383 | 0.794 |
|  | Delta_fALFF_C4_L_Precun | -0.039 | 0.883 | 0.967 |
|  | Delta_fALFF_C5_R_Temporal | 0.047 | 0.861 | 0.967 |
|  | Delta_fALFF_C6_R_Frontal | -0.250 | 0.337 | 0.794 |
|  | Delta_fALFF_C7_L_Caudate | -0.530 | 0.0292 | 0.234 |
| **pre_MD_Cingulum** | Delta_fALFF_C1_L_Cereb | 0.120 | 0.646 | 0.967 |
|  | Delta_fALFF_C2_L_Angular | 0.110 | 0.687 | 0.967 |
|  | Delta_fALFF_C3_R_Cereb | 0.450 | 0.071 | 0.352 |
|  | Delta_fALFF_C4_L_Precun | 0.120 | 0.646 | 0.967 |
|  | Delta_fALFF_C5_R_Temporal | -0.150 | 0.572 | 0.967 |
|  | Delta_fALFF_C6_R_Frontal | 0.420 | 0.0913 | 0.365 |
|  | **Delta_fALFF_C7_L_Caudate** | **0.750** | **0.00079** | **0.0167*** |
| **pre_MD_Uncinate** | Delta_fALFF_C1_L_Cereb | 0.037 | 0.891 | 0.967 |
|  | Delta_fALFF_C2_L_Angular | -0.086 | 0.744 | 0.967 |
|  | Delta_fALFF_C3_R_Cereb | 0.290 | 0.255 | 0.722 |
|  | Delta_fALFF_C4_L_Precun | 0.034 | 0.898 | 0.967 |
|  | Delta_fALFF_C5_R_Temporal | -0.100 | 0.694 | 0.967 |
|  | Delta_fALFF_C6_R_Frontal | 0.340 | 0.181 | 0.563 |
|  | Delta_fALFF_C7_L_Caudate | 0.640 | 0.00692 | 0.0969 |
| **pre_RD_Cingulum** | Delta_fALFF_C1_L_Cereb | 0.120 | 0.660 | 0.967 |
|  | Delta_fALFF_C2_L_Angular | 0.042 | 0.876 | 0.967 |
|  | Delta_fALFF_C3_R_Cereb | 0.380 | 0.131 | 0.458 |
|  | Delta_fALFF_C4_L_Precun | 0.059 | 0.824 | 0.967 |
|  | Delta_fALFF_C5_R_Temporal | -0.130 | 0.619 | 0.967 |
|  | Delta_fALFF_C6_R_Frontal | 0.460 | 0.063 | 0.352 |
|  | **Delta_fALFF_C7_L_Caudate** | **0.750** | **0.00079** | **0.0167*** |
| **pre_RD_Uncinate** | Delta_fALFF_C1_L_Cereb | -0.005 | 0.989 | 0.996 |
|  | Delta_fALFF_C2_L_Angular | -0.066 | 0.802 | 0.967 |
|  | Delta_fALFF_C3_R_Cereb | 0.230 | 0.383 | 0.794 |
|  | Delta_fALFF_C4_L_Precun | 0.044 | 0.869 | 0.967 |
|  | Delta_fALFF_C5_R_Temporal | -0.120 | 0.653 | 0.967 |
|  | Delta_fALFF_C6_R_Frontal | 0.270 | 0.290 | 0.773 |
|  | Delta_fALFF_C7_L_Caudate | 0.570 | 0.0191 | 0.178 |
| **pre_AD_Cingulum** | Delta_fALFF_C1_L_Cereb | 0.160 | 0.528 | 0.967 |
|  | Delta_fALFF_C2_L_Angular | 0.250 | 0.332 | 0.794 |
|  | Delta_fALFF_C3_R_Cereb | 0.480 | 0.053 | 0.330 |
|  | Delta_fALFF_C4_L_Precun | 0.230 | 0.372 | 0.794 |
|  | Delta_fALFF_C5_R_Temporal | -0.110 | 0.673 | 0.967 |
|  | Delta_fALFF_C6_R_Frontal | 0.340 | 0.181 | 0.563 |
|  | Delta_fALFF_C7_L_Caudate | 0.610 | 0.0104 | 0.116 |
| **pre_AD_Uncinate** | Delta_fALFF_C1_L_Cereb | -0.023 | 0.929 | 0.982 |
|  | Delta_fALFF_C2_L_Angular | 0.066 | 0.801 | 0.967 |
|  | Delta_fALFF_C3_R_Cereb | 0.440 | 0.0807 | 0.352 |
|  | Delta_fALFF_C4_L_Precun | 0.230 | 0.373 | 0.794 |
|  | Delta_fALFF_C5_R_Temporal | -0.140 | 0.589 | 0.967 |
|  | Delta_fALFF_C6_R_Frontal | 0.290 | 0.258 | 0.722 |
|  | Delta_fALFF_C7_L_Caudate | 0.520 | 0.0334 | 0.234 |

Spearman's rank correlations (N=17). *p*-values (FDR-corrected) were adjusted across all 56 comparisons (8 DTI metrics × 7 fALFF clusters). * = Significant. Abbreviations: DTI, Diffusion Tensor Imaging; fALFF, fractional amplitude of low-frequency fluctuation; FDR, False Discovery Rate; FA, Fractional Anisotropy; MD, Mean Diffusivity; RD, Radial Diffusivity; AD, Axial Diffusivity.
