# Supplementary material for: Central effects of short-term spinal cord stimulation in postherpetic neuralgia: a longitudinal fMRI and DTI study
Source: Front Neurosci. 2026 Jan 13;19:1744783. doi: 10.3389/fnins.2025.1744783 (PMC12835296; doi:10.3389/fnins.2025.1744783)
Supplement: Supplementary file 7 [file Table_7.DOCX]

**Supplementary Table S7.** Individual stSCS Parameters and Medication Usage.

| **Subjects ID** | **Age/Sex** | **stSCS Parameters** | | | **Medication Usage** | |
| --- | --- | --- | --- | --- | --- | --- |
|  |  | **Frequency**  **(Hz)** | **Pulse Width**  **(μs)** | **Amplitude**  **(mA)** | **Pregabalin**  **(mg/day)** | **Tramadol Use**  **(n, 50mg/inj)** |
| **Sub-01** | 70/M | 50 | 400-500 | 2.1-3.3 | 300 | 0 |
| **Sub-02** | 67/M | 50-60 | 300-500 | 1.6-2.8 | 300 | 0 |
| **Sub-03** | 62/M | 50 | 400 | 1.6-2.5 | 300 | 0 |
| **Sub-04** | 63/M | 60 | 350-500 | 2.8-4.6 | 300 | 0 |
| **Sub-05** | 65/M | 50 | 300-500 | 1.3-2.4 | 300 | 1 |
| **Sub-06** | 57/M | 50 | 400 | 1.2-1.6 | 300 | 2 |
| **Sub-07** | 43/F | 60 | 350-500 | 1.5-3.0 | 300 | 0 |
| **Sub-08** | 78/M | 40-50 | 300-450 | 2.4-3.4 | 150 | 0 |
| **Sub-09** | 79/F | 50 | 500 | 3.0-3.6 | 300 | 2 |
| **Sub-10** | 71/F | 50 | 450 | 3.0-4.2 | 300 | 2 |
| **Sub-11** | 73/M | 50-60 | 300-450 | 1.5-3.2 | 300 | 0 |
| **Sub-12** | 59/M | 60 | 350-400 | 1.1-2.3 | 300 | 0 |
| **Sub-13** | 63/M | 40 | 300-400 | 1.2-2.1 | 300 | 1 |
| **Sub-14** | 59/M | 50 | 400 | 3.1-4.8 | 300 | 0 |
| **Sub-15** | 80/M | 40-50 | 350-450 | 2.8-4.1 | 150 | 5 |
| **Sub-16** | 60/M | 50 | 300-450 | 1.3-2.7 | 600 | 3 |
| **Sub-17** | 61/F | 60 | 400 | 2.1-3.0 | 300 | 0 |

Stimulation parameters (Frequency, Pulse Width) are presented as a fixed value if maintained constant, or as a range if dynamically adjusted during the 14-day treatment. Current amplitude was titrated individually to maintain optimal paresthesia coverage. Tramadol was administered intramuscularly (50 mg/injection) as a rescue medication for breakthrough pain; values represent the total number of injections received. Abbreviations:stSCS, short-term spinal cord stimulation; M, Male; F, Female.
